# SMN Deficiency Destabilizes ABCA1 Expression in Human Fibroblasts: Novel Insights in Pathophysiology of Spinal Muscular Atrophy

**DOI:** 10.3390/ijms24032916

**Published:** 2023-02-02

**Authors:** Francesca Gabanella, Annalisa Onori, Cinzia Pisani, Marco Fiore, Giampiero Ferraguti, Andrea Colizza, Marco de Vincentiis, Marco Ceccanti, Maurizio Inghilleri, Nicoletta Corbi, Claudio Passananti, Maria Grazia Di Certo

**Affiliations:** 1 CNR-Institute of Biochemistry and Cell Biology, Department of Sense Organs, Sapienza University of Rome, Viale del Policlinico 155, 00161 Rome, Italy; 2 CNR-Institute of Molecular Biology and Pathology, Department of Molecular Medicine, Sapienza University of Rome, Viale Regina Elena 291, 00161 Rome, Italy; 3 Department of Experimental Medicine, Sapienza University of Rome, Viale del Policlinico 155, 00161 Rome, Italy; 4 Department of Sense Organs, Sapienza University of Rome, Viale del Policlinico 155, 00161 Rome, Italy; 5 Center for Rare Neuromuscular Diseases, Department of Human Neuroscience, Policlinico Umberto I, Sapienza University of Rome, 00185 Rome, Italy

**Keywords:** SMN, SMA, plasma membrane, ABCA1, cholesterol

## Abstract

The deficiency of survival motor neuron protein (SMN) causes spinal muscular atrophy (SMA), a rare neuromuscular disease that affects different organs. SMN is a key player in RNA metabolism regulation. An intriguing aspect of SMN function is its relationship with plasma membrane-associated proteins. Here, we provide a first demonstration that SMN affects the ATP-binding cassette transporter A1, (ABCA1), a membrane protein critically involved in cholesterol homeostasis. In human fibroblasts, we showed that SMN associates to ABCA1 mRNA, and impacts its subcellular distribution. Consistent with the central role of ABCA1 in the efflux of free cholesterol from cells, we observed a cholesterol accumulation in SMN-depleted human fibroblasts. These results were also confirmed in SMA type I patient-derived fibroblasts. These findings not only validate the intimate connection between SMN and plasma membrane-associated proteins, but also highlight a contribution of dysregulated cholesterol efflux in SMA pathophysiology.

## 1. Introduction

Survival motor neuron protein (SMN) influences the RNA life cycle and RNA-related pathways in all eukaryotic cells [1,2,3]. SMN is encoded by two almost identical genes, named *SMN1* and *SMN2*, located on chromosome 5q13 of the human genome. These genes differ by only five base pairs, remarkably, by a single nucleotide within the coding sequence (C > T). The centromeric *SMN2* gene primarily produces alternatively spliced transcripts that encode defective SMN. Consequently, *SMN2* cannot fully compensate for *SMN1* alterations (unless *SMN2* is present in multiple copies). Deletions or, less frequently, mutations of the telomeric *SMN1* gene, represent the diagnostic parameter of spinal muscular atrophy (SMA), one of the most common pediatric genetic diseases [4,5]. SMA is classified into four different subtypes (SMA type I–IV), based on age of onset and clinical severities. The spectrum of SMA also includes the type 0 variant that identifies cases in which the degenerative process can already be detected during late stages of pregnancy [6]. Degeneration of alpha-motoneurons in the spinal cord and a progressive muscle weakness, are the main clinical manifestations observed in the most severe form of the disease. Although the large alpha-motoneurons in the spinal cord display the highest susceptibility, SMN deficiency perturbs additional tissues and organs [7,8]. This is consistent with the fact that SMN drives multiple facets of RNA metabolism, ranging from transcription to local translation, in all cell types [2,3]. Our idea is that a large cell surface extension and polarity can exacerbate the adverse consequences of SMN deficiency. In this context, an intriguing aspect of SMN function may be its physical and functional link with plasma membrane (PM) compartments. In human fibroblasts, we showed that SMN binds to structural components of the PM, such as caveolin-1, and relocalizes at lipid raft microdomains during active cytoskeleton dynamics [9]. Interestingly, SMN is required to establish proper cell polarity by the accurate synchronization, in time and space, of actin filament polymerization and protein synthesis. Moreover, our previous studies reported that SMN affects membrane composition, as well as the association of distinct transcripts to the PM [10], which emerges as a docking site for RNA–protein complexes [10,11]. These findings prompted us to investigate if exists an implication of SMN in the composition and metabolism of the PM. Indeed, a study by Deguise and colleagues showed an increased propensity to developing dyslipidemia and liver steatosis in patients with SMA [12]. Notably, this research is in line with early studies identifying defects in fatty acid oxidation and lipid metabolism in SMA [13,14,15]. In the era of SMA therapy, a deep understanding of the molecular mechanisms underlying this severe pathology is crucial to provide more clinical benefits; furthermore, we focused on the intriguing relationship between SMN and PM networks.

Cholesterol is a major lipid constituent of the plasma membrane, contributing to membrane integrity and fluidity; it plays an essential role in several biological process [16,17,18]. Due to its unique ability to interact with phospholipids, cholesterol influences membrane microdomains, membrane trafficking, and signal transduction. Importantly, cholesterol can also interact with membrane proteins, affecting their function. Dysregulated cholesterol homeostasis is implicated in several human pathologies, including cancer, cardiovascular and neurodegenerative disease [19,20]. Mammalian cells have developed sophisticated mechanisms to prevent abnormal accumulation of cholesterol in cellular membranes [21].

The energy-dependent efflux of cholesterol from cells is mediated by ATP-binding cassette (ABC) transporters, primarily ABCA1. ABCA1 is a membrane-associated protein that acts to transfer intracellular cholesterol to apoliproteins (ApoA-I), promoting the formation of nascent high-density lipoproteins/HDLs [21,22,23]. Mutations in the *ABCA1* gene have been associated with Tangier disease, a rare genetic disorder characterized by extremely low levels of HDL and apoA-I; excessive deposition of cholesteryl esters in macrophages, and lipid deposits in cells including fibroblasts, Schwann cells, and myofibers [24,25,26,27]. *ABCA1* is expressed ubiquitously; however, in some tissues there is a discrepancy between *ABCA1* mRNA and protein expression, suggesting the existence of regulatory mechanisms at both transcriptional and post-transcriptional levels [28].

Here, we provide a first demonstration of a relationship between SMN and the membrane-associated protein ABCA1. In human fibroblasts, we showed that SMN associates to *ABCA1* mRNA, and affects its subcellular localization. Consistent with the pivotal role of ABCA1 in cholesterol efflux from cells, we visualized an accumulation of cholesterol in SMN-depleted cells, compared with the control. Importantly, this result was confirmed in SMA type I patient-derived fibroblasts. These findings further confirm an intimate connection between SMN function and plasma membrane dynamics, and point out that dysregulated cholesterol homeostasis may be a critical component in SMA pathophysiology.

## 2. Results

### 2.1. SMN Associates to ABCA1 mRNA

**Figure 1:**
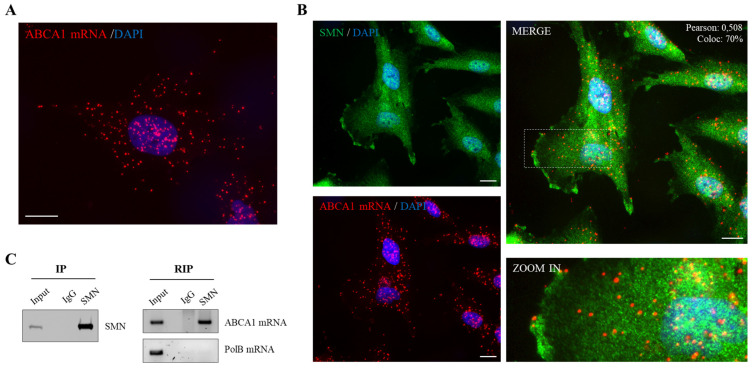
SMN associates to ABCA1 mRNA. (**A**) Representative image of human fibroblasts subjected to a padlock assay targeting ABCA1 mRNA. AlexaFluor 595-labelled probe allowed the detection of amplicons (red dots). Nuclei were labelled with DAPI (blue). Scale bars, 10 µm. (**B**) Fibroblasts were subjected to a combination of SMN immunostaining (green) and padlock assay targeting ABCA1 mRNA (red), and then were imaged via high-resolution epifluorescence microscopy. Nuclei were stained with DAPI (blue). Scale bar, 10 μm. Higher magnification of the boxed area (ZOOM IN) reveals some of the overlapped fluorescent signals (yellow dots). The classical Pearson coefficient of the pixel–intensity correlation and the percentage of ABCA1 mRNA (red) colocalizing with SMN (green) were reported in the MERGE panel. (**C**) Cellular extracts were processed for RNA-immunoprecipitation (RIP) assay using SMN monoclonal antibody-conjugated beads. As a negative control, mouse IgG-conjugated beads were used. Immunoblotting validating the efficiency of SMN immunoprecipitation (left panel). ABCA1 mRNA presence in RIP samples was checked semiquantitative RT-PCR (right panel). RT-PCR analysis of DNA Polymerase Beta (*PolB*) mRNA was used as negative control. Panels are representative of three independent experiments.

Keeping in mind that SMN associates and impacts structural components of the plasma membrane [9,10], we deeply explored this intriguing feature of SMN, starting to focus on ABCA1, a major regulator of cholesterol homeostasis. First, we analyzed the localization pattern of endogenous ABCA1 mRNA in single cells. We subjected human fibroblasts to padlock assay, a method useful to visualize transcripts of interest with high selectivity [10]. By fluorescence microscopy, ABCA1 mRNA appeared to be located throughout the cytoplasm and nuclear/perinuclear regions of the cell (Figure 1A). Interestingly, several fluorescent dots/amplicons were present at the cell periphery, indicating traffic to and/or from the plasma membrane. Next, we combined ABCA1 padlock probe and SMN immunostaining. As shown in Figure 1B, distinct subcellular sites showed the presence of overlapped fluorescent signals (yellow dots), suggesting a partial colocalization between ABCA1 mRNA and SMN. Most of the overlapped dots were detectable in nuclear/perinuclear sites. To note, some of these overlapped dots were present also in the cell periphery, in close proximity to the plasma membrane. To provide a demonstration of a physical association between SMN and ABCA1 mRNA, we carried out RNA-immunoprecipitation (RIP) experiments. In agreement with fluorescence microscopy data, we revealed the presence of SMN and ABCA1 mRNA in the same complex in human fibroblasts (Figure 1C). This novel interaction of SMN prompted us to suppose SMN implication in ABCA1 modulation.

### 2.2. SMN Knockdown Affects the Subcellular Distribution of ABCA1 mRNA

**Figure 2:**
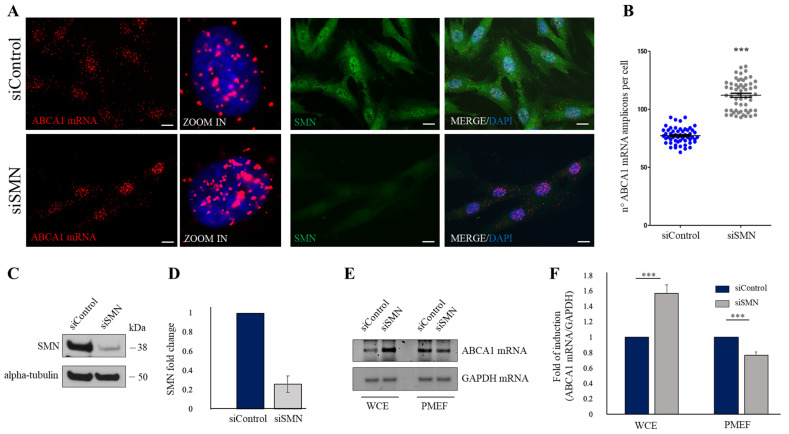
SMN knockdown affects the subcellular distribution of ABCA1 mRNA. (**A**) Padlock assay targeting ABCA1 mRNA (red dots). Images in ZOOM IN panels represent high magnifications of a selected cell. Nuclei were labelled with Dapi (blue). The combination of padlock assay targeting ABCA1 mRNA (red dots) and SMN immunostaining (green) was carried out in siControl- and siSMN-transfected human fibroblasts. Nuclei were stained with DAPI (blue). Scale bar, 10 μm. (**B**) Quantitative analysis of ABCA1 mRNA amplicons per cell (n = 20 cells were analyzed for each condition). Results from three independent experiments are plotted in the graph. Mean ± s.d. are illustrated. Asterisks indicate significant differences using unpaired *t*-test (*** *p* < 0.0001). (**C**) Western blot analysis validating SMN reduction in siSMN-transfected fibroblasts. Equal amounts of proteins were immunoblotted for the indicated antibodies. (**D**) Histogram indicates SMN fold change in siSMN-transfected fibroblasts compared to siControl, taken as 1. The means of three independent experiments are reported. Error bars represent s.d. (**E**) ABCA1 mRNA and glyceraldehyde-3-phosphate dehydrogenase (GAPDH) mRNA were checked in WCE and PMEF of siControl- and siSMN-transfected fibroblasts by semiquantitative RT-PCR, and analyzed by agarose gel electrophoresis. Panels are representative of three independent experiments. (**F**) Densitometric analysis of ABCA1 mRNA normalized to GAPDH mRNA in WCE and PMEF of siSMN-transfected fibroblasts compared with the control (siControl). The graph illustrates the means of three independent experiments. Error bars represent s.d. Asterisks indicate significant differences using unpaired *t*-test (*** *p* < 0.01).

The next step was to verify an implication of SMN in subcellular localization of ABCA1 mRNA. To this end, we down-regulated SMN levels by transfection of human fibroblasts with a pool of SMN1-selective siRNAs (siSMN). Scrambled siRNAs were used as control (siControl). Forty-eight hours after transfection, fibroblasts were subjected to a padlock assay targeting the ABCA1 mRNA. A combination of padlock assay and SMN immunostaining was carried out to identify SMN-depleted cells (Figure 2A). In siControl-transfected cells, ABCA1 mRNA was found diffusely distributed throughout the cytoplasm, in peripheral as well as nuclear/perinuclear sites (Figure 2A, siControl). Conversely, ABCA1 mRNA amplicons exhibited a propensity to accumulate at nuclear/perinuclear subregions upon SMN knockdown (Figure 2A siSMN). By quantitative analysis of amplicons, we observed a general significant increase in ABCA1 transcript in SMN-deficient cells (Figure 2B). Noteworthy, by padlock experiments we observed that SMN deficiency disturbed peripheral location of ABCA1 mRNA. Given our previous study, suggesting that SMN could promote membrane compartmentalization of a subset of transcripts [10], we wanted to verify the following: (1) whether a pool of ABCA1 mRNA could associate to plasma membrane compartments; (2) whether this association could occur in an SMN-dependent manner. To this end, we approached a biochemical method that was useful to isolate plasma membrane-enriched fractions (PMEFs) from cultured cells [9]. Both siSMN- and siControl-transfected fibroblasts were processed to obtain PMEFs. Then, total RNA was extracted from whole cell extracts (WCE) as well as their respective PMEFs. By a semiquantitative RT-PCR, we observed that ABCA1 transcript was detectable in WCE and, most importantly, in PMEFs of human fibroblasts (Figure 2C). Remarkably, we found that SMN knockdown reduced the abundance of ABCA1 mRNA in the PMEFs, despite its higher detection in whole cells (Figure 2D). Considering the assumption that the PM could act as a docking site for a subset of transcripts, we suggested the following: (1) ABCA1 mRNA may be one of the membrane-associated transcripts; (2) ABCA1 mRNA membrane compartmentalization may be influenced by SMN.

### 2.3. SMN Deficiency Down-Regulates ABCA1 Protein Levels and Causes Intracellular Accumulation of Cholesterol

**Figure 3:**
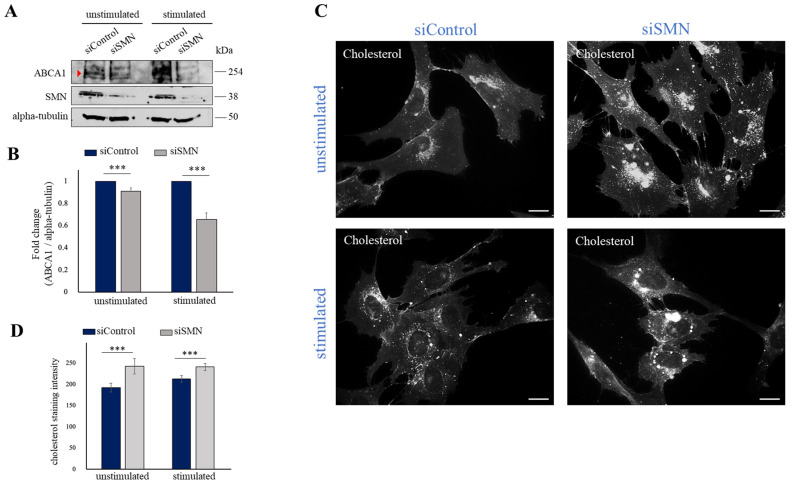
SMN deficiency down-regulates ABCA1 protein levels and causes intracellular accumulation of cholesterol. (**A**) Representative Western blot analysis of protein extracts from unstimulated and stimulated transfected cells. Equal amounts of proteins were blotted and checked for ABCA1 and SMN. Levels of alpha-tubulin were monitored as a control of protein loading. (**B**) Densitometric analysis of ABCA1 normalized to alpha-tubulin in unstimulated and stimulated siSMN transfected fibroblasts compared with the control (siControl). The graph illustrates the means of three independent experiments. Error bars represent s.d. Asterisks indicate significant differences using unpaired *t*-test (*** *p* < 0.01). (**C**) Representative images of free cholesterol in unstimulated and stimulated transfected human fibroblasts by filipin staining. Scale bars, 10 µm. (**D**) Cholesterol staining intensity analysis in siSMN-transfected fibroblasts compared with the control (siControl) in both unstimulated and stimulated conditions. The graph illustrates the means of cholesterol staining intensity determined by ImageJ 1.53a software. Error bars represent s.d. Asterisks indicate significant differences using unpaired *t*-test (*** *p* < 0.01).

**Figure 4:**
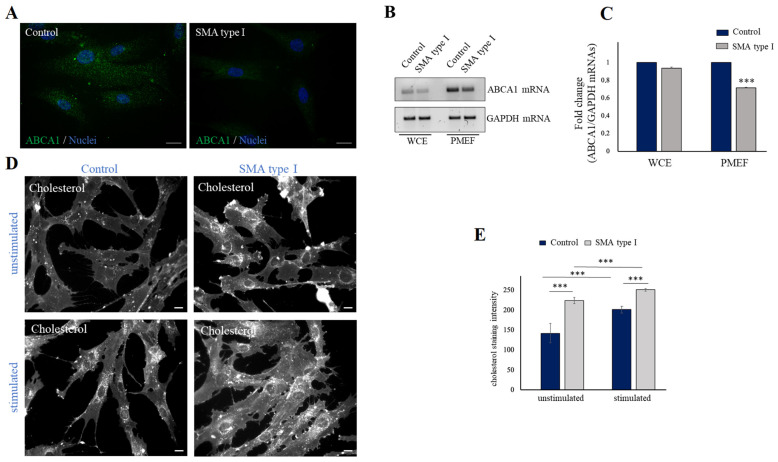
ABCA1 and intracellular distribution of cholesterol are altered in SMA type I fibroblasts. (**A**) Representative images of ABCA1 immunostaining (green) in control (unaffected) and SMA type I fibroblasts. Nuclei were stained with DAPI (blue). Scale bar, 10 μm. (**B**) *ABCA1* mRNA and *GAPDH* mRNA were checked in WCE and PMEF of control (unaffected) and SMA type I fibroblasts using semiquantitative RT-PCR. Panels are representative of three independent experiments. (**C**) Densitometric analysis of *ABCA1* mRNA normalized to *GAPDH* mRNA in WCE and PMEF of control and SMA type I fibroblasts. The graph illustrates the means of three independent experiments. Error bars represent s.d. Asterisks indicate significant differences using unpaired *t*-test (*** *p* < 0.01). (**D**) Representative images visualizing free cholesterol via filipin staining in unstimulated and stimulated SMA type I fibroblasts compared to primary fibroblasts from a healthy individual. Scale bars, 10 µm. (**E**) Cholesterol staining intensity analysis in SMA type I-derived fibroblasts compared to primary fibroblasts from a healthy individual (Control), in both unstimulated and stimulated conditions. The graph illustrates the means of cholesterol fluorescence intensity determined by ImageJ software. Error bars represent s.d. Asterisks indicate significant differences using unpaired *t*-test (*** *p* < 0.01).

Given the novel link between SMN and ABCA1 mRNA, we asked whether SMN could also influence expression levels of ABCA1 protein. Using Western blot analysis, we checked and compared ABCA1 protein content in siSMN- and siControl-transfected fibroblasts (Figure 3A). Furthermore, since ABCA1 protein induction occurs in an ATP-dependent manner [26], we also monitored ABCA1 protein levels in cells stimulated by a standard ATP-depletion/recovery assay [9,29,30]. A band of approximately 254 kDa, corresponding to the molecular weight of ABCA1, was detected in unstimulated fibroblasts. In our system, ABCA1 protein appeared unchanged or slightly reduced in SMN-depleted cells, compared to control (Figure 3A,B, unstimulated). Interestingly, ATP-depletion/recovery stimulation caused an increase in ABCA1 protein, which was impaired in SMN-depleted cells (Figure 3A,B, stimulated). Thus, our findings demonstrate that SMN impacts ABCA1 mRNA intracellular distribution, and this correlates with an altered ABCA1 protein level. Given the pivotal role of ABCA1 in counteracting cellular cholesterol accumulation, we suspected a disturbed distribution of cholesterol following SMN knockdown. To verify this hypothesis, we visualized intracellular cholesterol by subjecting fibroblasts to filipin staining. As shown in Figure 3C (unstimulated), images from fluorescence microscopy were indicative of abnormal accumulation of free cholesterol into siSMN-transfected fibroblasts, compared to siControl-transfected cells. This event persisted following ATP-depletion/recovery treatment (Figure 3C, stimulated). We also verified this issue in primary fibroblasts derived from an SMA type I patient. By a semiquantitative RT-PCR, we showed that despite the almost unchanged abundance of ABCA1 transcript in whole cell extracts, SMA type I fibroblasts exhibited a clear reduction in plasma membrane-associated ABCA1 transcript (Figure 4A,B). These findings were in line with the results obtained in siSMN-transfected fibroblasts. Next, we monitored free cholesterol distribution by filipin staining (Figure 4C, unstimulated). In SMA type I fibroblasts, we found an abnormal cholesterol accumulation compared to primary fibroblasts from a healthy individual (control) in the steady-state condition (unstimulated). In stimulated healthy control fibroblasts, we observed an increase in cholesterol staining that became more accumulated and clearly aberrant/disorganized in an SMN deficiency background (SMA type I) (Figure 4C, stimulated). Together, these findings provide a first demonstration that SMN associates to and affects ABCA1 mRNA, impacting cholesterol efflux regulation.

## 3. Discussion

SMA is a rare genetic disease whose complexity emerges by its pathological impact involving different organs, beyond the neuromuscular system. It has been reported that SMA patients exhibit an increased risk of dyslipidemia, suggesting an implication of SMN in lipid metabolism [12]. Although intriguing, this aspect of SMA pathophysiology remains to be addressed.

We recently showed a relationship between SMN and plasma membrane-related networks [9,10]. SMN not only associates to plasma membrane proteins, but also makes the PM competent to restrict protein synthesis at the subcellular level. Moreover, experimental evidence corroborates the concept that SMN may impact membrane trafficking and endocytosis pathways [3]. These findings suggest that SMN can promote the establishment of specialized subdomains by a fine synchronization of membrane remodeling and protein synthesis control. This implies that the amount of SMN that a cell needs for proper performance depend on the extension of its endomembrane system. As known, SMN binds ribosomal proteins as well as their coding transcripts [9,10,31]. Recently, Lauria and colleagues reported that SMN deficiency causes ribosome depletion at the beginning of the coding sequence of distinct mRNAs. Some of these SMN-specific mRNAs are linked to lipid metabolism, such as *SREBF1* transcript [31]. Notably, SREBF1 is a transcription factor that targets the promoter sequence of genes involved in cholesterol biosynthesis and lipid homeostasis [32]. In this framework, we wanted to establish whether SMN can impact lipid components of the plasma membrane. Since cholesterol is an essential structural constituent of the PM, we started to explore a relationship between SMN and ABCA1, a key regulator of cholesterol homeostasis. In human fibroblasts, we showed the existence of intracellular complexes in which SMN coexists with *ABCA1* transcript. Indeed, a co-traffic of SMN and *ABCA1* mRNA was also suggested by our imaging studies. As indicated by both biochemical and microscopy approaches, a pool of ABCA1 transcripts was prone to localize at the cell periphery, and to associate with the PM. Importantly, membrane compartmentalization of *ABCA1* mRNA was impaired by SMN deficiency, despite an overall increment in *ABCA1* mRNA levels. Accordingly, ABCA1 protein, whose expression levels increased in an activity-dependent context, appeared down-regulated in SMN-depleted stimulated cells. Other transcripts appeared to accumulate in nuclear regions of fibroblasts following SMN knockdown [10,33]. Indeed, mature mRNAs of several genes may be detected in the nucleus, independently of their expression levels [34,35]. It is plausible that nuclear retention of distinct transcripts may occur (1) when their subcellular trafficking is impaired; (2) to prevent aberrant translation processes. Regarding *ABCA1* mRNA, at this stage we can only speculate that SMN may work to properly promote the subcellular localization and translation of *ABCA1* mRNA.

One of downstream effects of the reduced expression levels of *ABCA1* is the cytoplasmatic accumulation of cholesterol, due to its efflux impairment [21]. Accordingly, in our system we demonstrated an aberrant distribution of cholesterol in siSMN-transfected fibroblasts and, most importantly, in SMA type I patient-derived fibroblasts. Thus, in an SMN deficiency background, the intracellular content of cholesterol appears dysregulated. Furthermore, since ABCA1 synergizes with ABCG1 in regulating reverse cholesterol transport [36], we cannot exclude a relationship between SMN and additional ATP-binding cassette (ABC) transporters.

It is noteworthy that ABCA1, in addition to regulating cholesterol efflux, modulates annexin A1 (ANXA1), which is associated with anti-inflammatory responses [37,38]. Interestingly, an important paralog of the *ANXA1* gene is *ANXA2*, whose transcript displays an SMN-dependent axonal localization [39]. An interesting notion is that miR-183 levels are increased in SMN-deficient neurons [40]. Remarkably, it has been demonstrated that miR-183 binds the 3’UTR of *ABCA1* mRNA, and negatively regulates its expression [41,42]. Collectively, these findings fit very well with the idea that SMN supports membrane dynamics by a fine-tuning of membrane-related factors. Homeostatic regulation of cholesterol is needed to remodel membrane platforms underlying specialized cellular activities. Notably, cholesterol biosynthesis and efflux are dysregulated in amyotrophic lateral sclerosis (ALS) [43], a neuromuscular pathology that shares several clinical aspects with SMA. It is important to mention that cholesterol stabilizes neuromuscular junctions (NMJs), promoting their maturation from patch- to pretzel-type morphology [44]. Moreover, it has been reported that proper cholesterol content ensures fine neuromuscular transmission and synaptic integrity [16]. This is a crucial issue in SMN-related networks, since NMJ dysfunction is an early event in SMA pathophysiology [45]. In this context, it is important to point out that cholesterol is essential for the membrane expansion of glial cells, and it is one of the main lipid molecules in myelin. Notably, in a mouse model of SMA, it has been shown that myelination defects as well as NMJ alterations may be reversed by selective restoration of SMN levels in myelinating Schwann cells [46]. Interestingly, ANXA1 can trigger Schwann proliferation and migration following peripheral nerve injury [47]. Given the relationship between ANXA1 and ABCA1, it is plausible to suppose a critical role of ABCA1 also in Schwann cells activity. Indeed, in Tangier families, a demyelinating multineuropathy condition has been described [48,49]. These findings highlight an intriguing interplay between SMN and ABCA1 networks. Thus, it will be interesting to deeply assess whether and how SMN influences cholesterol homeostasis and its related pathways.

In conclusion, in the era of SMA-modifying therapies [50], this study provides further understanding of the molecular landscape related to SMN functions, and can help to develop complementary approaches that provide more clinical benefits for patients.

## 4. Materials and Methods

### 4.1. Antibodies and Reagents

The following antibodies were used: anti-SMN mouse monoclonal antibody (cat. no. 610647, BD Transduction Laboratories; work dilution for Western blotting, 1:10,000; for immunofluorescence, 1:150); anti-ABC1 mouse monoclonal antibody (cat. no. sc-53482 Santa Cruz Biotechnology, Dallas, TX, USA; work dilution for Western blotting, 1:200); anti-alpha-tubulin mouse monoclonal antibody (cat. no. T6074, Sigma-Aldrich, St. Louis, MO, USA; work dilution for Western blotting, 1:2000). The secondary antibodies conjugated to horseradish peroxidase were purchased from Jackson ImmunoResearch Laboratories (West Grove, PA, USA) and used at a dilution of 1:5000. The Alexa Fluor488-conjugated secondary antibodies were purchased from Thermo Fisher (Waltham, MA, USA), and were used at a dilution of 1:200. Filipin (cat. no. F-9765) was purchased from Sigma-Aldrich.

### 4.2. Cell Cultures and Transfection

hTert-immortalized human fibroblasts, were grown in Dulbecco’s modified Eagle’s medium (DMEM, Gibco, Grand Island, NY, USA), supplemented with heat-inactivated 10% FBS (Australian) (Gibco), penicillin-streptomycin (Gibco) and GlutaMAX (Gibco). Human fibroblasts from SMA type I patient (GM00232) and healthy control (GM08333) were obtained from Coriell Institute for Medical Research (Camden, NJ, USA), and grown in DMEM medium supplemented with 10% FBS, penicillin/streptomycin, and GlutaMAX. All cell cultures were maintained at 37 °C in a humidified atmosphere of 5% CO_2_. For knockdown experiments, cells were transfected with Lipofectamine 2000 (Thermo Fisher Scientific) and a combination of three siRNA-27 duplexes targeting the human SMN1 gene (OriGene, Rockville, MD, USA), following manufacturer’s instructions. Universal scrambled siRNA duplex was used as negative control. Cells were harvested after 48 h post transfection.

### 4.3. ATP Depletion and Recovery Assay

The ATP depletion/recovery assay was performed as described previously [9,29]. Briefly, fibroblasts were incubated in PBS supplemented with 1 mM CaCl_2_, 1 mM MgCl_2_ and 20 mM NaN_3_, for 1 h. NaN_3_-containing buffer was then replaced with fresh medium supplemented with heat-inactivated 10% FBS (Australian) (Gibco) for 30 min, allowing ATP recovery.

### 4.4. Preparation of Plasma-Membrane-Enriched Fractions

Plasma-membrane-enriched fractions (PMEFs) were isolated as previously described [9]. Briefly, cells were lysed in buffer A (5 mM Tris-HCl pH 7.4, 1 mM EGTA, 1 mM DTT, and 320 mM sucrose). Extracts were passed through a 26G needle five times and centrifuged at 1000× *g* for 10 min at 4 °C. The supernatant was kept, and the pellet was quickly vortexed in the presence of the original volume of lysis buffer and centrifuged at 1000× *g* for 10 min at 4 °C. The two supernatants were pooled and centrifuged at 24,000× *g* for 20 min at 4 °C in a Beckman SW41 rotor. The supernatant was discarded, and the pellet was resuspended in 12 mL of buffer B (5 mM Tris-HCl pH 7.4, 1 mM EGTA, and 1 mM DTT), and centrifuged at 24,000× *g* for 30 min at 4 °C in a Beckman SW41 rotor. The supernatant was discarded. The pellet was aliquoted and processed for RNA extractions.

### 4.5. Immunofluorescence and Filipin Staining

Cells were fixed with 4% formaldehyde in PBS, permeabilized in 0.2% Nonidet P40 (Boehringer Mannheim, Mannheim, Germany) for 20 min, and blocked with 1% BSA in PBS at room temperature. Samples were incubated sequentially with the appropriate primary and secondary antibodies. Slides were mounted with ProLong with Dapi (Thermo Fisher Scientific), and examined with a conventional epifluorescence microscope (Olympus BX53; Milano, Italy). For free cholesterol staining by filipin, cells were fixed in 4% formaldehyde in PBS, incubated with 1.5 mg of glycine/mL PBS for 10 min at room temperature to quench the formaldehyde, and stained with 0.05 mg/mL in PBS filipin for two hours at room temperature. Slides were mounted with ProLong (Thermo Fisher Scientific) and examined using a conventional epifluorescence microscope (Olympus BX53; Milano, Italy), using excitation at 340–380 nm and emission at 385–470 nm. Images were captured with a SPOT RT3 camera, elaborated by IAS v.5.0.1 software (Biosistem ’82, Rome, Italy), and analyzed by ImageJ, National Institutes of Health, Bethesda, MD, USA 1.53a software.

### 4.6. RNA Immunoprecipitation (RIP) Assay

Cells were resuspended in IP Buffer (50 mM Tris-HCl pH 7.5, 250 mM NaCl, 5 mM EDTA, 50 mM NaF, 0.1 mM NaVO4, 0.1% Triton X-100, 5% glycerol, and complete protease inhibitor cocktail (Roche, Indianapolis, IN, USA)), in the presence of RNase inhibitors (Thermo Fisher Scientific). Extracts were vortexed 3 times for 10 s, incubated in ice for 20 min, and centrifuged at 10,000× *g* for 7 min at 4 °C. For the immunoprecipitation assay, the protein lysate was pre-cleared with Protein A/G-Agarose beads (Roche, Indianapolis, IN, USA), pre-saturated in 2% BSA-PBS by replacing beads 3 times within 90 min, at 4 °C. Then, 750 µg of extract was immunoprecipitated in IP buffer overnight with the anti-SMN monoclonal antibody. As negative control, the immunoprecipitation was carried out with mouse IgG-beads. The beads were washed five times for 5 min at 4 °C in IP buffer, and once in PBS buffer. The immunoprecipitated samples were resuspended in IP buffer. A portion of immunoprecipitation was analyzed with Western blot analysis. RNA was extracted using TRIzol^®^ reagent (Thermo Fisher Scientific), according to the manufacturer’s instructions. RNAs were converted to cDNAs using a High Capacity cDNA Reverse Transcription kit (Thermo Fisher Scientific).

### 4.7. Western Blot Analysis

All samples were processed in sample buffer and incubated at 100 °C for 10 min, except samples designated for ABCA1 detection. For ABCA1 detection, samples were incubated with sample buffer containing beta-mercaptoethanol at room temperature for 30 min. Protein extracts were electrophoresed through standard 6% SDS-PAGE, and transferred onto nitrocellulose membranes (GE Healthcare; Milano, Italy). Immunodetection of the reactive bands was revealed by chemiluminescence (ECL kit, GE Healthcare), and analyzed by iBright 1500 (Thermo Fisher Scientific Inc.).

### 4.8. RNA Extraction, Retrotranscription and Semiquantitative Real-Time PCR (RT-PCR)

RNA from whole cell extract and PMEF fraction was extracted using TRIzol^®^ reagent according to the manufacturer’s instructions, and was then reverse transcribed using a High Capacity cDNA Reverse Transcription kit (Thermo Fisher Scientific). A semiquantitative PCR (RT-PCR) assay was performed in triplicate using the BioMix 2× (Bioline) according to the manufacturer’s instructions. The primer sequences used in this study are shown in Table 1.

### 4.9. Padlock Assay

Phosphorylation of the padlock probe and padlock assay were performed as previously described [9].

### 4.10. Quantification and Statistical Analysis

All experiments were performed on at least three independent biological replicates. Data are presented as mean ± s.d. Statistical analysis was performed using GraphPad Prism 9.4.1 software. Data were analyzed using an unpaired *t*-test; *p* < 0.01 was considered statistically significant.

## Figures and Tables

**Table 1:** Oligos used in the study.

Primer Name	Primer Sequence (5′-3′)
RT-PCR GAPDH F	CATGAGAAGTATGACAACAGCCT
RT-PCR GAPDH R	AGTCCTTCCACGATACCAAAGT
RT-PCR POLB F	GTGAGACAAAGTTCATGGGTGT
RT-PCR POLB R	GTGAAACCCTTTTCTAGGGCAT
RT-PCR ABCA1 F	TACATCTCCCTTCCCGAGCA
RT-PCR ABCA1 R	GGAGCTGGAGCTGTTCACAT
Padlock Probe ABCA1	CATGTCACTCCAGCTTTTTTTTCTCAATTCTGCTACTTTACTACCTCAATTCTGCTACTGTACTACTTTTTTCATCACCTCCTGTCG
RCA Primer	AGTACAGTAGCAGAATTGAG
AlexaFluor 595-labelled probe	CTCAATTCTGCTACTTTACTAC

## Data Availability

The authors confirm that the data supporting the findings of this study are available within the article.
